# Chemo-hormone therapy of non-well-differentiated endocrine tumours from different anatomic sites with cisplatinum, etoposide and slow release lanreotide formulation

**DOI:** 10.1038/sj.bjc.6603734

**Published:** 2007-04-17

**Authors:** P Correale, A Sciandivasci, C Intrivici, A Pascucci, M T Del Vecchio, S Marsili, V Savelli, L Voltolini, M Di Bisceglie, A Guarnieri, G Gotti, G Francini

**Affiliations:** 1 Medical Oncology, Department of Human Pathology and Oncology, Siena University School of Medicine, Viale Bracci 11, 53100, Siena, Italy; 2 Medical Pathology Section, Department of Human Pathology and Oncology, Siena University School of Medicine, Viale Bracci 11, 53100, Siena, Italy; 3 Second Division of General Surgery, Siena University School of Medicine, Viale Bracci 11, 53100, Siena, Italy; 4 Division of Thoracic Surgery, Siena University School of Medicine, Viale Bracci 11, 53100, Siena, Italy

**Keywords:** neuroendocrine tumours, chemotherapy, hormonal manipulation

## Abstract

We report the results of a phase II trial in patients with metastatic endocrine tumours from different sites, which aimed to evaluate the anti-tumour activity and toxicity of a cisplatinum and etoposide regimen administered in combination with the somatostatin agonist lanreotide given in slow release formulation. Between January 1999 and November 2003, 27 patients with histological diagnoses of endocrine tumours with different degrees of differentiation, excluding well differentiated carcinoid neoplasms, received intravenous (i.v.) administration of cisplatinum (30 mg m^−2^) and etoposide (100 mg m^−2^) on days 1–3 and intramuscular administration of 60 mg lanreotide on day 1, in a 21-day cycle. All of the patients were evaluable for toxicity and response. The treatment was very well tolerated as no grade 4 toxicity was observed. Four patients achieved a complete response, six a partial response, 12 experienced disease stabilisation and five disease progression. The average time to progression and to survival were 9 and 24 months respectively. These results suggest that this chemo-hormone therapy regimen is well tolerated and active in patients with non-well differentiated endocrine tumours.

Non-differentiated endocrine tumours represent a heterogeneous and controversial group of neoplastic diseases, whose existence has been largely underestimated, as they are often reported as mixed, undifferentiated, or anaplastic malignancies (Buchanan *et al*, 1986; Moertel, 1987; Greco and Hainsworth, 2005; Jensen and Doherty, 2005). These tumours may arise from any organ or tissue undertaking neuroendocrine control, such as respiratory, gastroenteric, and urinary tracts, as well as secretory glands like the prostate, breast, and pancreas, and usually present common phenotypic and functional features (Buchanan *et al*, 1986; Moertel, 1987; Greco and Hainsworth, 2005; Jensen and Doherty, 2005).

Neuroendocrine cells derive from the same multi-potent stem cells that are responsible for either cutaneous or mucosal tissue replacement. On the basis of their original genotypic programme, and in response to specific environmental stimuli, these multi-potent cells may differentiate in somatic epithelial cells (glandular, cutaneous, or mucosal cells) or cells with neurosecretive potential and neurovegetative control capability (Buchanan *et al*, 1986; Moertel, 1987; Langley, 1994; True, 2004; Gordon *et al*, 2005; Long *et al*, 2005; Sauer *et al*, 2006).

Similarly, tumour cells with neuroendocrine phenotype derive from stem cells genetically altered to progress into cancer (through inherited and/or acquired mutations). In this case, the derivative cells may dynamically undergo a caricatural differentiation that may resemble epithelial, glandular, or neuroendocrine cells. On the basis of different kinds and levels of molecular and genetic alterations, these cells may follow distinct differentiation pathways and may stop their distinct differentiation programmes at different stages of maturation (Buchanan *et al*, 1986; Hansson and Abrahamsson, 2003; Wright *et al*, 2003; Bishop, 2005; Long *et al*, 2005). Respiratory, gastroenteric, and urinary tracts, as well as the prostate, are physiologically under strict neurovegetative control, and so it is not surprising that the majority of neuroendocrine tumours arise in these anatomic sites. Neuroendocrine tumours may manifest at different degrees of differentiation, from well-differentiated (carcinoids) to poorly differentiated, or anaplastic or somatic/neuroendocrine mixed forms. In some cases, they may be very difficult to recognise because there is no single marker to identify an undifferentiated neuroendocrine tumour; so, diagnosis must rely on the correct interpretation of pathological data (histology, immunohistochemistry, and sometimes electronic microscopy), biohumoral studies, blood/urinary tests, biological behaviour of the neoplasia, the natural history and the progression of the disease, and patients' symptoms (Wiedenmann and Huttner, 1989; Polak, 1993; Nicholson and Ryan, 2000; De Lellis, 2001; Bishop, 2005). Neuroendocrine tumours may arise with clinically different modalities and signs (paraneoplastic syndromes), which are often related to the different degrees of biological aggressiveness and to the different levels of production of specific hormones and peptides (Buchanan *et al*, 1986; Moertel, 1987). When well differentiated, they retain a low level of local and metastatic aggressiveness. However, they often give rise to endocrine syndromes related to the inappropriate production of peptides and amines with different hormonal profiles. These include neuron-specific enolase (NSE), 5-hydroxytryptamine (5-HT), 5-hydroxytryptophan (5-HTP), synaptophysin, chromogranins A and C, other peptides such as insulin, growth hormone, neurotensin, adrenocorticotropic hormone (ACTH), *β*-melanocyte-stimulating hormone, gastrin, pancreatic polypeptide, calcitonin, substance P, various other tachykinins (neuropeptide K), growth hormone–releasing hormone (GHRH), bombesin, and various growth factors such as transforming growth factor (TGF)-*β*, platelet-derived growth factor (PDGF), and fibroblast growth factor (FGF)-*β* (Fenoglio-Preiser, 2001; Oberg, 2002), many of which exert a powerful functional activity.

The less differentiated, anaplastic, and mixed forms are considered to be much more aggressive than the well-differentiated forms and are believed to be much more aggressive than their epithelial and glandular counterparts. Similar to small cell lung cancer, which belongs to this family of neoplasms, they are much more responsive to specific anti-cancer treatments and are very sensitive to platinum-based polychemotherapy (Mitry *et al*, 1999; Mitry and Rougier, 2001; Singhal *et al*, 2006).

The possibility of a phenotypic switch of advanced tumours from adenocarcinoma (mainly of the prostate and pancreas) to a neuroendocrine phenotype has also been shown, and this fact correlates with enhanced sensitivity to several cytotoxic drugs (Mitry *et al*, 1999; Mitry and Rougier, 2001; Hainsworth *et al*, 2006; Singhal *et al*, 2006). In this context, we have shown in a previous study that drug-resistant colon cancer cells, driven to neuroendocrine differentiation following exposure to phorbol myristate acetate *in vitro*, lose both the epithelial phenotype and their (type I) multidrug-resistant phenotype, becoming highly sensitive to topoisomerase II inhibitors such as adriamycin and etoposide (Correale *et al*, 1994). The recognition of undifferentiated or somatic/neuroendocrine mixed forms of endocrine tumours could therefore have very important prognostic and therapeutic implications.

Sensitivity to somatostatin analogues (SSAs) is another characteristic aspect of these neoplasms. Tumour cells with neuroendocrine differentiation vary in their expression of functional somatostatin receptors (SSTR), whose engagement and stimulation with SSAs may produce efficacious cytostatic effects. Somatostatin (SST) binding to SSTRs is known to be capable of interfering with the production and release of many different classes of hormones and growth factors (such as GH, IGF, VEGF) and to be able to transmit a direct anti-proliferative message. Sensitivity to somatostatin analoguess, alone or in combination with interferon *α* have been used to treat well-differentiated and moderately well-differentiated endocrine tumours and to control the carcinoid syndrome (related to the inappropriate production of molecules with hormonal activity) that is often associated with these tumours (Kvols *et al*, 1986; Lamberts, 1999).

Considering this background, we hypothesised that the therapeutic use of SSAs such as lanreotide and octreotide, associated with an efficacious polychemotherapy regimen, may represent an active treatment for aggressive endocrine tumours and mixed forms.

We therefore designed a phase II trial involving patients with metastatic non-well-differentiated endocrine tumours deriving from different anatomic sites, which aimed to evaluate the antitumour activity and toxicity of a novel chemohormonal-therapy regimen that combines a cytotoxic polychemotherapy with (i.v.) cisplatinum (CDDP) and etoposide with the long lasting release formulation of lanreotide SSA.

## PATIENTS AND METHODS

The study protocol was approved by our local Ethics Committee, and was performed in accordance with the good clinical practice (GCP) guidelines. All patients gave their written informed consent. The study involved 27 patients with histological diagnosis of non-well differentiated and mixed endocrine tumours arising in different anatomic sites (Table 1). All of the patients were at an advanced stage of disease and all had an ECOG performance status of ⩽2 and a life expectancy of ⩾3 months. To be enrolled in the study, the patients had to have normal renal and hepatic function, a white blood cell (WBC) count of >2500 mm^−3^, haemoglobin levels of >9 mg mm^−3^, a platelet cell count of >90 000 mm^−3^, and a cardiac ejection fraction of >46%. The exclusion criteria were: well-differentiated carcinoid tumours; poor performance status (ECOG⩾3); severe valvular and wall motion abnormalities or cardiac failure; arrhythmia, central nervous system (CNS) metastases; secondary malignant tumours; signs of active hepatitis or liver failure; chronic or acute renal failure; active infectious disease; or a history of other severe cardiovascular disease.

### Study design

The phase II study was prospectively planned according to Simon's two-stage minimax design to test the hypothesis that our new chemo-hormone therapy schedule combining CDDP, etoposide, and lanreotide is an active treatment for patients with non-well differentiated neuroendocrine tumours and mixed forms.

The minimax two-stage procedure was designed to test a null hypothesis of *P*⩽0.150 *vs* an alternative of *P*⩾0.350, with an expected sample size of 20.15 and a probability of early termination of 0.604. In these conditions, if the combination is not considered to be active, there is only a 0.046 probability (4.6%) of discharging an active treatment (the target for this value was 0.050); conversely, if the regimen is found to be active, there is a 0.197 probability (19.7%) that it is actually not active (the target for this value was 0.200). The objective response rate (CR+PR) was the primary end point for the statistical analysis, while the disease control rate and the time to progression were secondary end points. For this study, we selected a 15% response rate as a null hypothesis and a 35% response rate as an alternative hypothesis, with a 0.05*α*-error and a 0.20*β*-error. In this case, the treatment under investigation should be considered inactive if less than 2 responses are recorded out of 15 consecutive patients in the first series and fewer than 7 responses out of 28 patients in the whole series (Hintze, 2004). We considered the regimen as active when a response rate of 35% was recorded, considering that the trial did not exclude patients receiving second-line treatment. Furthermore, the study was designed to involve patients with non-well-differentiated endocrine tumours, whose histological analysis showed different levels of neuroendocrine differentiation, and was not limited to the small cell and anaplastic forms that are highly sensitive to platinum-based polychemotherapy regimens.

### Patient treatment

Twenty-seven patients with non-well-differentiated endocrine tumours were enrolled in the study and gave their written informed consent, and received treatment with i.v. CDDP (30 mg m^−2^ days 1–3), i.v. etoposide (100 mg m^−2^ days 1–3) and i.m. lanreotide given as a long-lasting release formulation (60 mg day 1) in a 21-day cycle. Standard premedication with mannitol, corticosteroids, anti-emetic, and gastroprotective drugs was given to all patients before cytotoxic drug administration.

### Baseline and on-treatment clinical assessments

Before treatment, a complete medical history was taken of all patients who also underwent physical examination, a complete blood count, serum chemistry tests, and complete disease staging by means of chest X-rays, brain, chest, and abdominal computed tomography (CT), and liver and pelvic ultrasound. Considering that patients with endocrine tumours may be affected by paraneoplastic cardiopulmonary alterations (paraneoplastic fibrosis), an ultrasound investigation and ventricular function and pulmonary volume analysis were performed on all patients. The staging examinations were repeated every 2 months, whereas full blood counts, biochemistry profile, liver function tests, electrocardiography (ECG), chest X-rays, and urine analysis were performed weekly.

### Toxicity and response criteria

All eligible patients were evaluated for survival and toxicity, and they were considered evaluable for response when they had completed three treatment cycles. If the patients responded or had stable disease, the treatment was continued until the occurrence of disease progression or unacceptable toxicity. Overall survival was measured from the date of diagnosis to the date of death or the date of the last follow-up examination. Time to progression was evaluated from the beginning of treatment to the demonstration of disease progression or the date of the last follow-up examination. Response and toxicity were assessed using standard WHO criteria. A complete response was defined as the complete disappearance of all known measurable disease for at least 1 month, while a partial response was defined as a decrease of almost 50% in known lesions lasting for at least 1 month. The area of two-dimensional lesions was defined as the product of the longest diameter multiplied by the greatest perpendicular diameter; disease stabilisation was defined as a <50% decrease or <25% increase in evaluable lesions lasting for 1 month without the appearance of new lesions, and progressive disease was defined as a >25% increase in known disease or the appearance of new lesions (WHO criteria).

## RESULTS

### Demographics

Twenty-seven patients with a histological diagnosis of metastatic non-well-differentiated endocrine tumours were enrolled in the study and received treatment between January 1999 and November 2003. There were 22 men and five women, with an average of 63.5 years of age. Twenty-three of them presented at least one sign of a typical or atypical carcinoid syndrome at diagnosis. Twelve of them showed liver metastases, whereas 13 had previously undergone surgery and six had previously received a 5-fluorouracil based line of chemotherapy. Other characteristics are shown in Table 1. No patients with a pathological diagnosis of anaplastic malignancy, well-differentiated endocrine tumour or localised carcinoid tumour were enrolled in the study.

### Toxicity

A median of 20 weeks of treatment was administered per patient (range 15–24). The treatment was very well tolerated as no grade 4 toxicity was observed. No patients died during the treatment. Grade III haematological toxicity was the most common adverse event. Febrile neutropenia and anaemia were both reversible with the administration of specific growth factors. Thrombocytopenia, on the other hand, delayed treatment by 1 week in 25% of the cases. All patients were able to receive full doses of CDDP and etoposide for the entire treatment programme. No cases of grade III-IV diarrhoea, mucositis, oliguria, hypotension, or transaminase elevation were observed during the treatment (Table 2).

### Response

This study was designed with the intention to treat thus, all the patients were taken in consideration (Table 3). We observed a 37% objective response rate (four complete and six partial) and 81.5% disease control rate (10 objective responses+12 disease stabilisations). Only five patients experienced rapid tumour progression. The signs of carcinoid syndrome were completely reversed in all patients within the first week of treatment, which appeared to be associated with significant reduction of the urinary levels of HT, 5-HTP, and indoleacetic acid detected at baseline (data not shown). All responsive patients (OR+s.d.) showed a significant reduction in seric chromogranin A, with blood levels dropping from 65 (±27) to 15 (±12) U l^−1^ (*P*<0.05).

In this study, we recorded a very long median time to progression and relative survival of 9 months (95% CI=3–36), and 24 months (95% CI=10–36) respectively. The average follow-up of these patients was 44 months. Six patients, who achieved an objective response or showed disease stabilisation, were alive 48 months from enrolment. Among these six patients, the primary tumour site was unknown (two cases), or derived from the pancreas, lung, prostate or thyroid (one case each). With the exception of the last patient, who was diagnosed with a medullary thyroid carcinoma on secondary pathological revision, all of the others had a histological diagnosis of undifferentiated endocrine tumour.

## DISCUSSION

The results of our study suggest that a regimen based on the combined use of standard polychemotherapy and hormonal manipulation is a safe and active treatment for these patients. The regimen was considered active in the treatment of these malignancies, as a 37% objective response rate and a very high disease control and symptom control rate were recorded. Many of these patients had received a previous line of chemotherapy, and some of them had histological features that were different from small cell and other anaplastic forms, which are known to be very sensitive to platinum-based polychemotherapy. We also recorded a very long time to progression and survival — results that are perfectly in line with the study's rationale. The addition of an SAA to this chemotherapy regimen is based on the knowledge that SSTR 2, 3, 4, and 5 stimulation might have cytostatic effects on the tumour cells that survived the cytotoxic drugs, thus preventing or delaying their inter-cycle recovery. On this basis, we did not expect the chemo-hormonal combination to significantly enhance the tumour shrinking ability of the cytotoxic drugs, but we hypothesised its ability to enhance the rate of disease stabilisation in these patients, eventually prolonging their time to progression and survival.

Several trials have investigated the effects of chemotherapy, SAAs (either octreotide on lanreotide), and biological response modifiers such as *α*-interferon in the treatment of these tumours, achieving conflicting results in term of response rate, symptom control, and survival (Kvols *et al*, 1986; Moertel *et al*, 1991; Di Bartolomeo *et al*, 1995; Jensen, 1997; Bajetta *et al*, 1998, 2000, 2003, 2005; Rougier and Ducreux, 1999; Jensen and Doherty, 2001; Mitry and Rougier, 2001; Oberg, 2002; Faiss *et al*, 2003; Hainsworth *et al*, 2006), mainly due to the very difficult classification of these tumours and pathological analysis. Although equally able to control hormonal symptoms by reducing the secretion of biological amines and various peptides, these analogues exert a poor tumoricidal effect, being able to decrease tumour size in less than 15% of patients. However, SAAs possess a powerful tumorostatic effect, being capable of stabilising the growth of metastatic disease and prolonging survival (Jensen, 1997; Patel, 1997; Dierdorf, 2003; Faiss *et al*, 2003).

In this context, none has yet evaluated the possibility of combining cytotoxic chemotherapy with the administration of SSAs. We designed this regimen hypothesising that the administration of SAAs between two subsequent administration cycles of chemotherapy could sensitise tumour cells with neuroendocrine differentiation to the apoptotic effect of many different cytotoxic drugs, including CDDP and etoposide, and could contribute to delaying the recovery of drug-resistant or less sensitive cancer cells that usually occurs in the long (21–28 days) inter-cycle resting period. We also speculated that the metronomic use of SSAs may also synchronise the cell cycle of the tumour cells, thus making them a much more sensitive target for the cycle-specific cytotoxic drugs, enabling them to kill a greater fraction of in-cycle tumour cells. Preclinical models also suggest an anti-angiogenetic effect of SSAs that is believed to be able to reduce tumour production and release of VEGF (Kumar *et al*, 2004) that could synergise with the cytotoxic effects of chemotherapy. Finally, we considered that SSA administration could improve tolerance to cytotoxic drugs by possibly reducing the occurrence of gastroenteric toxicity (Low, 2004; Arabi *et al*, 2006). Currently only two SSAs are commercially available for clinical use in Europe: octreotide and lanreotide. Both were designed to bind the SSTR-2, whose stimulation of neuroendocrine cells is believed to inhibit the secretion of hormones and bioactive molecules, and they are currently considered to be equivalent. The results of preclinical studies also suggest that the SSA binding to SSTR-4 and -5 mediates a powerful cytostatic activity, while SSTR-3 activates a pro-apoptotic pathway (Li *et al*, 2005; Kvols and Woltering, 2006; Ruan *et al*, 2006). For our study, we chose lanreotide over octreotide, considering that there is no difference in SSTR-2-binding affinity and little difference in SSTR-3 and SSTR-5 binding, while only lanreotide is able to bind the cytostatic SSTR-4 (Patel, 1997). We believe that more promising results will be obtained when more selective SST analogues become available. In conclusion, we believe that the results of this study provide the rationale to carry out a randomised multicentre phase III trial to compare the efficacy of our chemo-hormonal combination *vs* polychemotherapy with CDDP and etoposide for the treatment of patients with non-well differentiated endocrine tumours.

## Figures and Tables

**Table 1:** Demographics

**Characteristics**	**No. of patients**
Patients evaluable for response	27
Patients evaluable for toxicity	27
	
*Age (years)*	
Median	63.5
Range	47–78
	
*Sex*	
Male	22
Female	5
Performance status (ECOG)	0–3
	
*Primary tumour*	
Lung	7
Thyroid	2
Gut	8
Pancreas	2
Prostate	4
Unknown	4
Previous surgery	13
Previous systemic treatment	4
None	5
One or more line of previous therapy	2
Carcinoid syndrome	23
	
*Disease extension*	
Stage IV	27
(A) Liver involvement	12
(B) No liver involvement	15

**Table 2:** Toxicity

**Grade III and IV toxicity episodes (27 patients)**	**No. of events**
Total courses	135
Thrombocytopenia	15
Anaemia	14
Neutropenia	25
Infection	4
Fever	16
Malaise	3
Pulmonary distress	1
Nausea/vomiting	1
Diarrhoea	0
Oliguria (<80 cc/8 h)	0
Creatinine (>8 mg/dl)	0
Arrhythmia	0
Hypotension	0
CNS-level of conscience	0
CNS-orientation	0
Hyperbilirubinemia	0

**Table 3:** Treatment response

**Clinical evaluation (cohort of 27 patients)**	**Number of patients**
(A) Complete response	4 (14.8%)
(B) Partial response	6 (22.2%)
(C) Stable disease	12 (44.4%)
Progressive disease	5 (18.5%)
Objective response rate (A+B)	10 (37.0%)
Disease control rate (A+B+C)	22 (81.5%)
Total number of cycles administered	135
Average number of cycles per patient	5 (range 3–12)
Time to progression	9 (range 3–36)
Overall survival	24 (range 10–36)
